# Characterization, Function, and Transcriptional Profiling Analysis of 3-Hydroxy-3-methylglutaryl-CoA Synthase Gene (*GbHMGS1*) towards Stresses and Exogenous Hormone Treatments in *Ginkgo biloba*

**DOI:** 10.3390/molecules22101706

**Published:** 2017-10-12

**Authors:** Xiangxiang Meng, Qiling Song, Jiabao Ye, Lanlan Wang, Feng Xu

**Affiliations:** College of Horticulture and Gardening, Yangtze University, Jingzhou 434025, Hubei, China; dreamer9212@163.com (X.M.); song70@126.com (Q.S.); yejiabao13@163.com (J.Y.); wangll30@126.com (L.W.)

**Keywords:** *Ginkgo biloba*, terpene trilactones, 3-hydroxy-3-methylglutaryl-CoA synthase, enzyme activity, functional complementation, expression profiling

## Abstract

3-Hydroxy-3-methylglutaryl-CoA synthase (HMGS) is one of the rate-limiting enzymes in the mevalonate pathway as it catalyzes the condensation of acetoacetyl-CoA to form 3-hydroxy-3-methylglutaryl-CoA. In this study, A *HMGS* gene (designated as *GbHMGS1*) was cloned from *Ginkgo biloba* for the first time. *GbHMGS1* contained a 1422-bp open-reading frame encoding 474 amino acids. Comparative and bioinformatics analysis revealed that GbHMGS1 was extensively homologous to HMGSs from other plant species. Phylogenetic analysis indicated that the GbHMGS1 belonged to the plant HMGS superfamily, sharing a common evolutionary ancestor with other HMGSs, and had a further relationship with other gymnosperm species. The yeast complement assay of GbHMGS1 in *HMGS*-deficient *Saccharomyces cerevisiae* strain YSC6274 demonstrated that *GbHMGS1* gene encodes a functional HMGS enzyme. The recombinant protein of GbHMGS1 was successfully expressed in *E. coli*. The in vitro enzyme activity assay showed that the *k*_cat_ and *K*_m_ values of GbHMGS1 were 195.4 min^−1^ and 689 μM, respectively. *GbHMGS1* was constitutively expressed in all tested tissues, including the roots, stems, leaves, female flowers, male flowers and fruits. The transcript accumulation for *GbHMGS1* was highest in the leaves. Expression profiling analyses revealed that *GbHMGS1* expression was induced by abiotic stresses (ultraviolet B and cold) and hormone treatments (salicylic acid, methyl jasmonate, and ethephon) in *G. biloba*, indicating that *GbHMGS1* gene was involved in the response to environmental stresses and plant hormones.

## 1. Introduction

*Ginkgo biloba* L., commonly called the maidenhair tree, is the oldest relict plant species in the world and is considered a “living fossil” [1]. Many bioactive constituents in the leaf extract of *G. biloba* are beneficial to human health; these components include flavonoids and terpene trilactones (TTLs including ginkgolides and bilobalide), which are proven to reduce blood pressure, dilate the peripheral blood vessels, discourage clot formation, promote blood circulation and cerebral metabolism, reinforce the walls of the capillaries, and protect nerve cells from harm during oxygen deprivation [2]. Flavonoids are the preferred natural medications for the treatment of dementia and can be obtained from many other plants, whereas TTLs are unique components of *G. biloba* [3] that play a key role in the active ingredients. Ginkgolide B has a specific antagonistic effect on platelet-activating factor [4], whereas bilobalide has a protective function against neuronal damage and can be used to treat demyelinating brain, spinal cord, and neurological diseases [5,6]. However, the dry leaves of *G. biloba* have very low content of TTLs [7]. The unique chemical structure of TTLs renders them difficult to synthesize through chemical and biological methods, therefore increasing TTL content in *G. biloba* is of important significance in the ginkgo industry. Many studies have shown that the key to increasing the content of TTLs is to increase the expression level of the key genes in their metabolic pathways [8,9,10].

**Figure 1:**
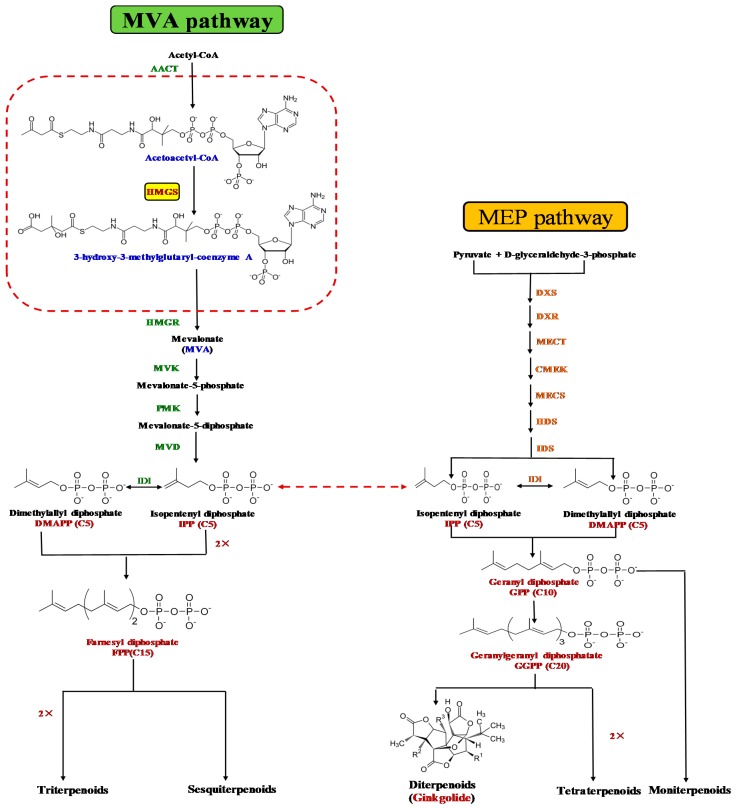
MVA and MEP pathways for terpenoid biosynthesis in *Ginkgo biloba*.

Terpenoids possess multiple ecophysiological functions, such as regulating plant growth and development, resisting various stresses, and providing direct and indirect plant defenses [11]. Considering their diverse functions, TTLs have been extensively applied as important raw materials in medicine, food, cosmetics, spices, and health care [12]. The terpenoids of plants, including TTLs, are synthesized mainly by two pathways: the classical cytosolic mevalonate (MVA) pathway, which facilitates the synthesis of sesquiterpenoids, triterpenoids, and steroids; and the plastidial methyl-erythritol-4-phosphate (MEP) pathway, which is MVA-independent and normally facilitates the formation of monoterpenoids, diterpenoids, and tetraterpenoids [13]. As a sesquiterpene, bilobalide is thought to be derived from farnesyl diphosphate (FPP) in the MVA pathway, but more authors suggested that bilobalide is most likely a product of partially degraded ginkgolide [14], whereas ginkgolide biosynthesis is thought to be achieved by the MEP pathway. However, a cross-talk seemed to occur between the two biosynthetic pathways because the universal five carbon skeleton–isopentenyl diphosphate (IPP) of terpenoid biosynthesis is derived from both MVA and MEP pathways [15]. As the second catalyzing enzyme, 3-hydroxy-3-methylglutaryl coenzyme-A synthase (HMGS) catalyzes the irreversible conversion of acetoacetyl-CoA into 3-hydroxy-3-methylglutaryl-CoA (HMG-CoA), which is the committed step in the MVA pathway of terpenoid biosynthesis in plants. 3-Hydroxy-3-methylglutaryl-CoA reductase (HMGR) catalyzes HMG-CoA to MVA and further converts it into isopentenyl pyrophosphate (IPP) and its isomer dimethylallyl pyrophosphate (DMAPP), which are the common precursors of many types of terpenoid biosynthesis. Afterward, IPP and DMAPP as the substrates are catalyzed by their corresponding enzymes to synthesize the following intermediates: geranyl diphosphate, FPP, and geranylgeranyl diphosphate, which are further catalyzed by the corresponding terpene synthase to produce all kinds of terpenoids [16,17] (Figure 1). Accumulated evidence suggests that HMGS plays an important role in the plant defense mechanism by participating in the MVA pathway, and the synthesis of plant terpenoids also positively correlates with the activity of HMGS. For example, a previous study reported that a positive correlation exists between HMGS activity and the dry rubber content of the latex from rubber trees [18]. Ren et al. [19] also stated that the ganoderic acid content was approximately 15.1%–24.2% higher than that of the control group, whereas the *GlHMGS* gene was overexpressed in *Ganoderma lucidum*. Another transgenic study also indicated that the overexpression of *BjHMGS* in *Arabidopsis thaliana* increases sterol concentration and enhances *Botrytis cinerea* resistance [20]. Therefore, we speculated that the *GbHMGS1* gene may play an important regulatory role in the resistance mechanism to abiotic stresses as well as in the biosynthesis of sesquiterpenoids (e.g., farnesene and bisabolene) in *G. biloba* [21].

Each gene in the MEP and MVA pathways that contributes to the biosynthesis of isoprenoid should be identified and characterized to determine the overall biosynthetic pathway of terpenoids in *G. biloba*. To date, many genes involved in the MEP pathways are isolated from *G. biloba*, such as 1-deoxy-d-xylulose 5-phosphate synthase (DXS) [22], 1-deoxy-d-xylulose 5-phosphate reducto-isomerase [23], 2-*C*-methyl-d-erythritol 4-phosphate cytidyltransferase [24], 4-(cytidine 5’-diphospho)-2-*C*-methyl-d-erythritol kinase (CMK) [8], 2-*C*-methyl-d-erythritiol 2,4-cyclo-diphosphate synthase [25], 1-hydroxy-2-methyl-2-(*E*)-butenyl-4-diphosphate synthase [26], and 1-hydroxy-2-methyl-2-(*E*)-butenyl-4-diphosphate reductase [27]. However, the presence of IPP cross-talk further complicates the origin of TTL building block in *G. biloba* [28]. Therefore, the MVA pathway may also have a minimal contribution to the biosynthesis of TTLs. Up to now, some of genes encoding enzymes involved in MVA pathway have been cloned and characterized from *G. biloba*, including *HMGR* [29], mevalonate diphosphate decarboxylase (MVD) [10], mevalonate kinase (MVK) and acetyl-CoA *C*-acetyltransferase (AACT) [30]. However, the *HMGS* genes in *G. biloba* are poorly studied. In this study, we cloned a *HMGS* gene (*GbHMGS1*) from *G. biloba* for the first time. The *GbHMGS1* gene was characterized by prokaryotic expression, and yeast complement assay were performed to confirm the function of *GbHMGS1*. Furthermore, the expression profiling of *GbHMGS1* in different tissues and under abiotic stresses (cold and ultraviolet B (UV-B)) and hormonal treatments (salicylic acid (SA), methyl jasmonate (MJ), and ethephon (Eth)) were investigated by qRT-PCR analysis. These findings will be useful supplements for unveiling the overall biosynthetic pathway as well as for the large-scale production of isoprenoid with pharmacological properties of this living fossil.

## 2. Results

### 2.1. Identification and Characterization of GbHMGS1

The conserved sequence composed of 542 bp was obtained by PCR using primers designed from the homologous regions of the reported HMGS proteins. Based on this sequence, a putative *HMGS* gene was obtained from *G. biloba* by RACE method. The full-length cDNA sequence of *GbHMGS1* was 1945 bp and contained a 1422-bp ORF encoding a 474 amino acid protein. A 98 bp 5′-UTR was upstream of the start codon, and the coding region was followed by a 425 bp 3′-UTR downstream from the stop codon (Supplementary Material Figure S1). A BLASTn search on NCBI showed that the cDNA sequence of *GbHMGS1* was highly similar to those of *HMGS* genes from other plants, indicating that the cloned gene was a member of the *HMGS* family. Therefore, the gene was designated as *GbHMGS1* (GenBank accession number: MF281039).

### 2.2. Bioinformatic Analysis of GbHMGS1 Protein

ExPASy online (http://web.expasy.org/compute_pi/) analysis results showed that the pI and theoretical molecular weight of the GbHMGS1 protein were 5.23 and 52.67 kDa, respectively, which are similar to those of previously reported plant HMGSs [31,32,33,34]. A BLASTp search in GenBank database (https://blast.ncbi. nlm.nih.gov) showed that deduced GbHMGS1 protein exhibited 74%–85% similarity to HMGS proteins of many other plants (Supplementary Material Table S2); specifically, 85%, 83%, 79%, 78%, 77%, 75% and 74% similarity to the counterparts of *Taxus media*, *Pinus sylvestris*, *Narcissus tazetta*, *Sorghum bicolor*, *Theobroma cacao*, *Hevea brasiliensis*, and *Camptotheca acuminata*, respectively, which further verified that GbHMGS1 belonged to plant HMGS family.

**Figure 2:**
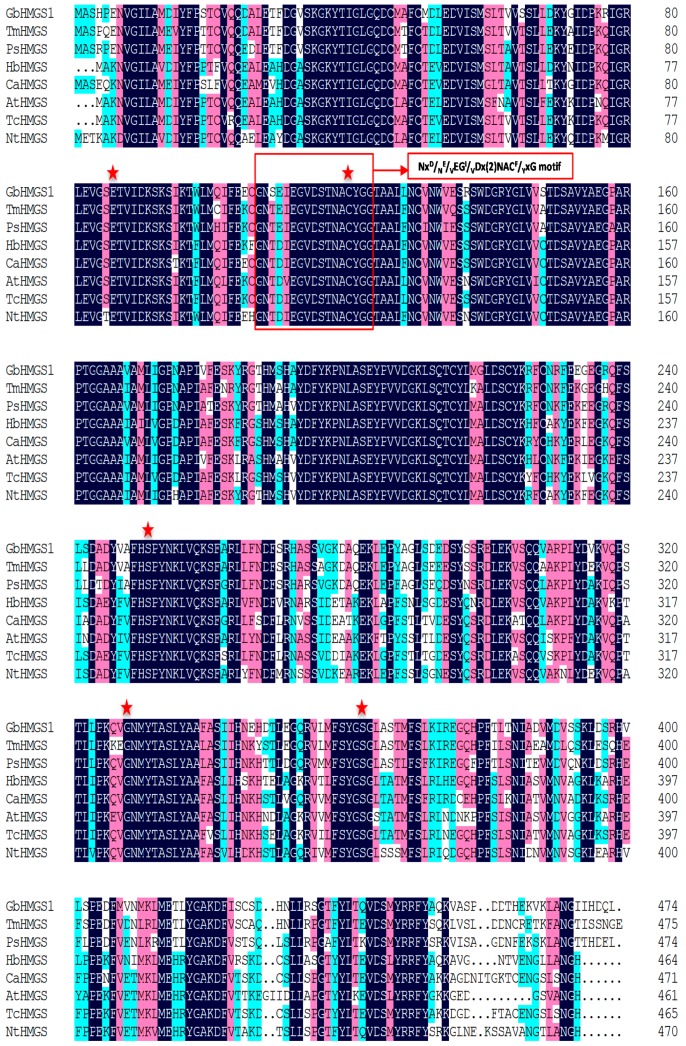
The multiple alignments of GbHMGS1 amino acid sequence with other HMGS proteins. The conservative motif, “NxD/NE/VEGI/VDx(2)NACF/YxG” is boxed. The active sites are indicated with asterisk. The species, protein names and GenBank accession numbers are as follows: *G. biloba* (GbHMGS1, MF281039); *T. media* (TmHMGS, AAT73206.1); *P. sylvestris* (PsHMGS, CAA65250.1); *H. brasiliensis* (HbHMGS, AAK73854.1); *C. acuminate* (CaHMGS, ACD87446.1); *A. thaliana* (AtHMGS, CAA58763.1); *T. cacao* (TcHMGS, XP_007040101.2); *N. tazetta* (NtHMGS, AHF81872.1). Dark blue: identity = 100%; red: 75% ≤ identity < 100%; light blue: 50% ≤ identity < 75%.

The multiple alignments of the deduced GbHMGS1 protein sequences with other HMGSs using software Vector NTI11.5 revealed that GbHMGS1 was highly homologous to HMGSs from other plants. Further sequencing analysis showed that GbHMGS1 protein contained the conserved motif “NxD/NE/VEGI/VDx(2)NACF/YxG” (Figure 2, marked with a red box) and five conserved sites (amino acids Glu86, Cys120, Ser251, Gly328, and Ser362) (Figure 2, marked with “asterisk”), which are crucial for HMGS activity [35]. These results indicated that GbHMGS1 catalyzes a similar enzymatic reaction to other plant HMGSs.

**Figure 3:**
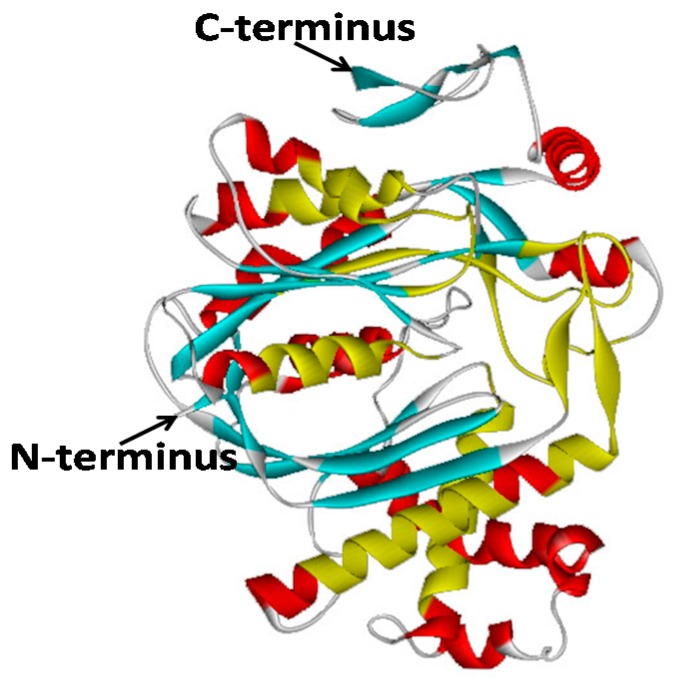
The three-dimensional model of GbHMGS1. The helices are indicated by helices in red and conservative area are indicated by helices in yellow. The sheets are indicated by patches in blue and yellow. Turns and loops are indicated by lines.

A comparative modeling of the 3D structure of GbHMGS1 (Figure 3) was generated based on the highest query coverage of the template *Brassica juncea* HMGS (2f82.1.A) with SWISS-MODEL [36] to further elucidate the GbHMGS1 protein. Similar to those of other HMGSs, the predicted 3D model of GbHMGS1 consisted of two structural regions called the lower and upper regions [35]. In addition, the interface of the upper and lower regions defined the acetoacetyl-CoA-binding sites [37], and all the conserved motifs and active sites were localized in a five-layered core structure of the upper region.

**Figure 4:**
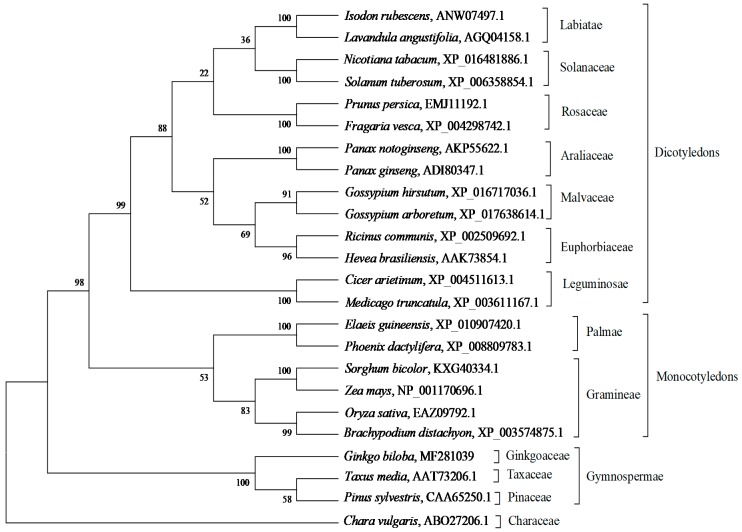
Phylogenetic tree of HMGSs from different species using the neighbor-joining method. The reliability of the tree is measured by bootstrap analysis with 1000 replications. The bars represent evolutionary distance.

A phylogenetic tree was constructed using the amino acid sequences of GbHMGS1 and HMGS protein sequences from other species to investigate the sequence diversity and evolutionary relationships among HMGS proteins. As shown in Figure 4, the phylogenetic tree was divided into four branches of Monocotyledons, Dicotyledons, Gymnospermae, and Algae. GbHMGS1 belonged to the Gymnospermae cluster and had the closest relationship with HMGSs from *T. media* and *P. sylvestris*, and they all belong to the Gymnospermae, which is in accordance with the species classification of plants. These relationships reflect the evolutionary conservation and evolutionary diversity of plant HMGS.

### 2.3. Prokaryotic Expression and In Vitro Enzyme Activity Analysis of GbHMGS1

To express *GbHMGS1* in *E. coli*, we constructed the pET32a-GbHMGS1 expression vector with the T7 promoter and a His-tag by ligating the coding region of *GbHMGS1* into pET-32a(+). The expression construct was evaluated for in-frame fusion through restriction enzyme digestion and DNA sequencing. Undergoing IPGE induction, GbHMGS1 was expressed as a major protein product in the insoluble, soluble and total cellular protein (Supplementary Material Figure S2). The molecular weight of the expressed recombinant protein with the His-tag was estimated to be about 52.67 kDa, coinciding with the molecular weight of GbHMGS1 protein predicted through bioinformatics. Purification of the recombinant GbHMGS1 protein was achieved by Nickel-CL agarose affinity chromatography and used for enzyme activity assay. The rates of catalysis by purified GbHMGS1 were measured as a function of acetoacetyl-CoA concentrations to evaluate the kinetic parameters of GbHMGS1. The initial velocity *V*_0_ plotted against substrate concentration yielded a rectangular hyperbola with *V*_max_ = 37.1 ± 5.4 nM·min^−1^ (Table 1). Sirinupong et al. [38] reported *K*_m_ = 530 ± 50 μM and a specific activity of 41.3 μmol·min^−1^·mg^−1^ using acetoacetyl-CoA as substrate for a recombinant HMGS from *H. brasiliensis*. Similar to HbHMGS, the purified GbHMGS1 had a higher *K*_m_ of 689 μΜ and a lower specific activity of 15.5 μmol·min^−1^·mg^−1^, indicating that GbHMGS1 possesses HMGS activity.

### 2.4. Functional Complementation of GbHMGS1 in Saccharomyces cerevisiae

**Figure 5:**
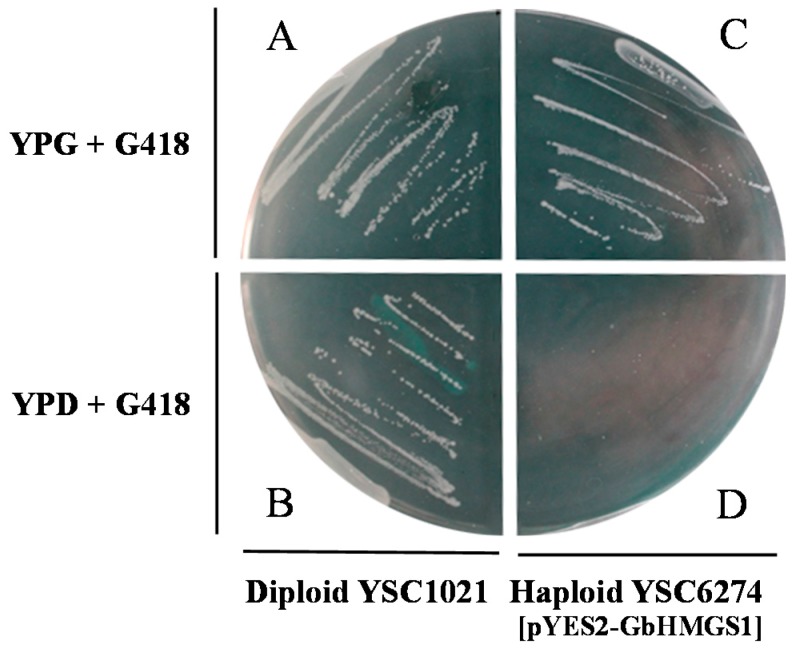
Functional complementation for the growth of the yeast strain YSC6274 complemented with GbHMGS1. (**A**) Diploid YSC1021 strain on YPG + G418 medium grew within 2 days; (**B**) Diploid YSC1021 strain on YPD + G418 medium grew within 2 days; (**C**) Haploid YSC6274 strain containing pYES2-GbHMGS1 on YPG + G418 medium grew within 2 days; (**D**) Haploid YSC6274 strain containing pYES2-GbHMGS1 on YPD + G418 medium failed to grow.

To determine the function of *GbHMGS1*, we successfully constructed the expression vector pYES2-GbHMGS1. The *GbHMGS1* cDNA driven by a galactose-dependent promoter was expressed in the HMGS-deficient haploid yeast strain YSC6274, and the mevalonate derived from MVA pathway is essential for yeast survival. Wild type *S. cerevisiae* strain YSC1020 could grow on either induction medium YPG with G418 (Figure 5A) or non-induction medium YPD with G418 (Figure 5B). By contrast, the transformed yeast YSC6274 strain harboring pYES2-GbHMGS1 could grow on YPG expression medium (Figure 5C) but not on the non-induction medium YPD with G418 (Figure 5D). The results showed that the expression of *GbHMGS1* can rescue the functional defect of the *hmgs* knockout yeast by encoding the HMGS enzyme to catalyze the synthesis of mevalonic acid. This data further confirmed that GbHMGS1 have HMGS activity.

### 2.5. Differential Expression Profiles of GbHMGS1 in Various Tissues of G. biloba

**Figure 6:**
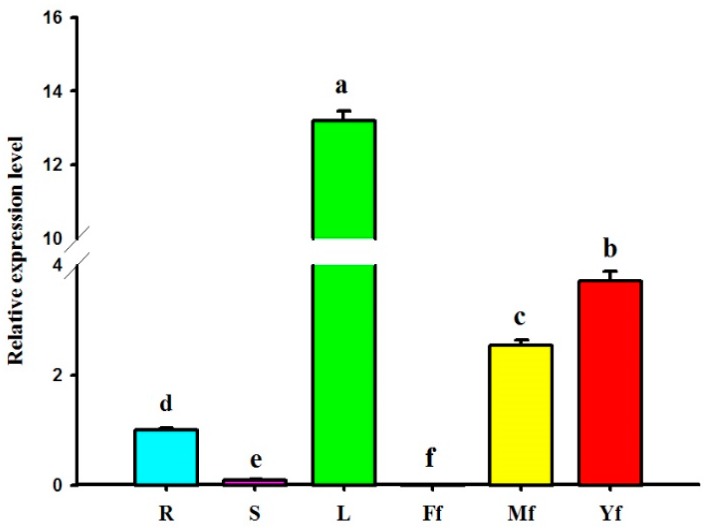
Spatial expression analysis of *GbHMGS1* in various tissues of *G. biloba*. R: root; S: stem; L: leaf; Ff: female flower; Mf: male flower; Yf: young fruit. The gene expression level of *GbHMGS1* in root was set to 1. Data are mean ± SD from triplicate experiments (*n* = 3). Means with different letters are significantly different at *p* < 0.05.

As shown in Figure 6, the *GbHMGS1* gene was constitutively expressed in all the tested tissues of *G. biloba*. In addition, *GbHMGS1* was highly expressed in the mature leaves, followed by young fruits, male flowers, and roots, but was lowly expressed in the stems and hardly in the female flowers. These results revealed that *GbHMGS1* had a preferential expression pattern in mature leaves.

### 2.6. GbHMGS1 Transcript Level and TTL Content Changes in G. biloba under the Induction of UV-B, Cold, MJ, Eth, SA Elicitor

**Figure 7:**
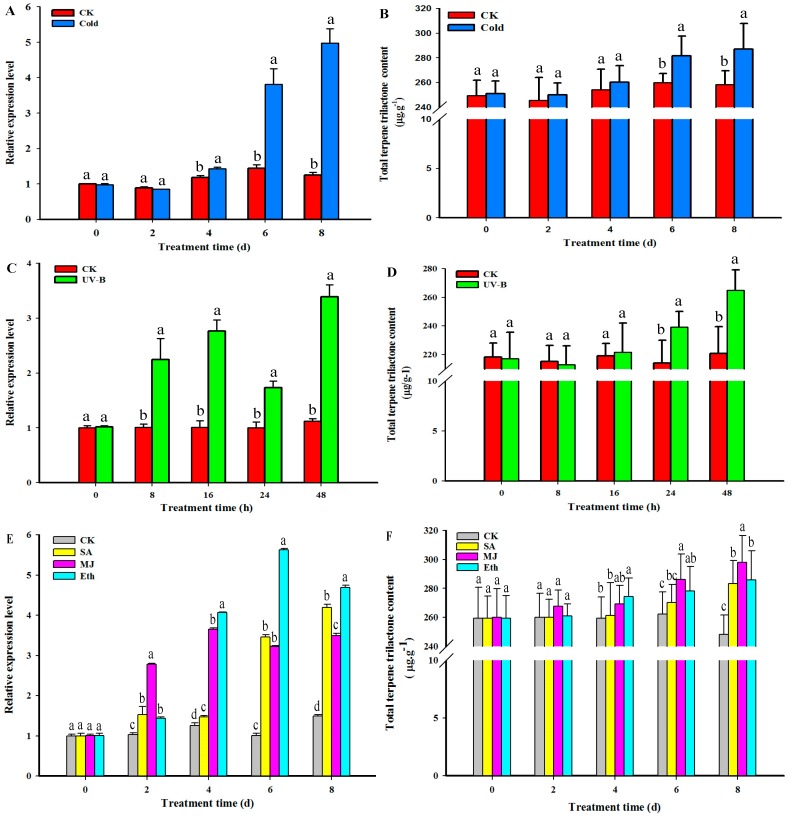
*GbHMGS1* transcript level and total terpene trilactone (TTL) content changes in *G. biloba* under the induction of Cold, UV-B, SA, MJ, Eth. (**A**) *GbHMGS1* transcript level changes by Cold; (**B**) TTL content changes by Cold; (**C**) *GbHMGS1* transcript level changes by UV-B; (**D**) TTL content changes by UV-B; (**E**) *GbHMGS1* transcript level changes by SA, MJ and Eth; (**F**) TTL content changes by SA, MJ and Eth. The expression levels were normalized to *Gb18S* gene. Data were analyzed as expression ratios relative to the level of control (CK). Data are mean ± SD from triplicate experiments (*n* = 3). Means with different letters from each time of post-treatment are significantly different at *p* < 0.05.

A time-course expression analysis of the *GbHMGS1* gene and TTL content under cold conditions, UV-B, MJ, Eth, and SA treatments was conducted on the ginkgo seedlings at 4–5 leaf stages to explore the roles of the *GbHMGS1* genes in response to different abiotic stresses and hormonal treatments. As shown in Figure 7, all elicitor inductions significantly increased *GbHMGS1* expression, and the TTL content was significantly enhanced after the corresponding various abiotic stresses and hormone treatments. Under UV-B, the transcript level of *GbHMGS1* increased gradually until 16 h post-treatment and decreased at 24 h, however, the level was still significantly higher than that of the control. Afterward, the level gradually increased again, *GbHMGS1* transcripts peaked at 48 h time point with about 2-fold increase compared with that of the control (Figure 7C). The TTL content was continuously induced and increased by 20% compared with that of the control after treatment for 48 h (Figure 7D). When exposed to the cold treatment, the expression level of the *GbHMGS1* gene was slightly down-regulated during short-term cold treatment and dropped 3.4% lower than that of the control 2 days after treatment. However, when the treatment time was extended, the expression level was up-regulated and significantly increased to about 3.98-fold of the control for up to 8 days of post-treatment (Figure 7A). The TTL content did not change significantly in the initial period of treatment but still on an upward trend, and peaked at 8 days (11.16% compared with that of the control) (Figure 7B).

In response to the exogenous hormone treatments, Figure 7F shows that TTL content all continuously increased with the increasing treatment time and reached maximum at 8 days after MJ, SA, and Eth treatments, with about 20%, 14.14% and 15% increase than that of the control, respectively. When exposed to the Eth treatment (Figure 7E), the transcript level of *GbHMGS1* was up-regulated within 6 days with the highest level of 458.79%, and then decreased over time. Figure 7E showed that the exogenous application of MJ caused a rapid increase in *GbHMGS1* transcripts, a significant increase in *GbHMGS1* expression at 4 days of post-treatment by 189.77%, a slight decrease thereafter, and a gradual increase again until the 8th day. The *GbHMGS1* expression was effectively induced by SA (Figure 7E), the transcript level of *GbHMGS1* continuously increased with the increasing treatment time and reached the highest level (181.61% as compared with that of the control) at 8 days of post-treatment.

## 3. Discussion

As a major rate-limiting enzyme in the terpenoid biosynthesis, HMGS converts acetoacetyl-CoA into HMGS-CoA in the MVA pathway. Furthermore, the *HMGS* gene family is related to the development and defense mechanism of plants [39]. Given its importance in MVA biosynthesis and stress resistance, HMGS received increasing attention and has been widely studied in angiosperms at the chemical, enzymological, and genetic levels [40,41,42]. However, few reports were found on HMGS enzymes or genes in gymnosperm species at the molecular level. The characterization and functional analysis of *GbHMGS1* will be helpful to further understand its role in terpenoid biosynthesis in *G. biloba*.

### 3.1. GbHMGS1 is a Member of HMGS Gene Family

In the present study, we successfully cloned a *HMGS* gene from *G. biloba* and analyzed its function through in vitro activity assay and yeast complement assay. GbHMGS1 protein contained 474 amino acids and weighed 52.67 kDa; this finding coincides with the result of the SDS-PAGE profile in prokaryotic expression and verifies that the plant HMGS protein is generally composed of 460–500 amino acid residues and has a relative molecular mass of 50–60 kDa [31,32,33,34]. Multiple alignments showed that the deduced GbHMGS1 was highly identical to other plant HMGSs and has the highest homology with *T. media* because both plants belong to Gymnosperms, which was speculated to have similar HMGS functions. GbHMGS1 contained the conserved substrate binding motifs of HMGSs “NxD/NE/VEGI/VDx(2)NACF/YxG” and five active sites (amino acids Glu86, Cys120, Ser251, Gly328 and Ser362). The conserved motif “NxD/NE/VEGI/VDx(2)NACF/YxG” forms the core of the enzyme structure, which is localized at the entrance of the active site, and controls the catalysis of substrates by HMGS. Mutation of this motif reduces the catalytic activity of the enzyme or leads to the formation of abnormal products, which is conserved across species [35]. In addition, three conserved amino acid residues, namely, Cys^120^, His^250^ and Asn^329^ (residue numbering corresponds to GbHMGS1) were known to be active residues essential for the catalytic activity in HMGS [43]. The polypeptide chain of GbHMGS1 also contains three domains, namely, N-terminus, catalytic region, and C-terminus. The N-terminus contains a conservative signal peptide sequence and shows a high degree of similarity across most plants. The C-terminus contains an important catalytic cysteine residue that acts as a nucleophile in the first step of reaction to accelerate the formation of HMG-CoA [37]. HMGS proteins also have highly similar 3D structures consisting of the upper and lower regions. These findings have deepened our understanding of the correlation between the structure and function of GbHMGS1, which also provides important basic data for further studying the metabolic process of terpenoids in *G. biloba*. In vitro enzyme activity assay verified that GbHMGS1 possesses HMGS activity. Complement assay revealed that the expression of *GbHMGS1* supplies the basic material for yeast survival, thereby confirming the catalytic function of GbHMGS1. Taken together, the results mentioned above suggested that *GbHMGS*1-encoded HMGS enzyme is a member of the plant HMGS protein family and has a similar catalytic activity of catalyzing the conversion of acetoacetyl-CoA into HMG-CoA in the MVA pathway in *G. biloba*.

### 3.2. Expression Pattern of GbHMGS1 in Different Tissues of G. biloba

The most unique components of *G. biloba* extracts are TTLs. Therefore, researchers have focused their studies on the TTLs of *G. biloba*. Strong evidence suggested that TTLs are synthesized in the roots and subsequently translocated to aerial parts through the phloem; the leaves only act as a storage location for ginkgolides and bilobalide [9,44,45]. Despite this progress, very little is known concerning the biosynthesis and identification of sesquiterpenes in *G. biloba*. Recently, two genes encoding (*E*,*E*)-farnesol and α-bisabolene synthases involved in sesquiterpene were cloned and functionally characterized from *G. biloba*. In future studies, whether the spatial expression profile of *GbHMGS1* is positively correlated with other sesquiterpenes (e.g., farnesene and bisabolene) [21] content in different tissues of *G. biloba* will be investigated in light of the fact that HMGS played an important role in sesquiterpene biosynthesis.

Up to now, tissue-dependent differences in *HMGS* expression level have also been studied for many medicinal plants. The expression pattern of *HMGS* in plant tissues greatly varies across different plants. For example, *HMGS* is constitutively expressed, strongest in the leaves, moderate in the stems, and weakest in the roots of *S. miltiorrhiza* and *C. acuminata* [31,32]. In addition, *TwHMGS* exhibited the highest expression level in the stem, followed by the leaf, and the expression level in the root was the lowest [33]. A common feature is that the constitutive high expression level of *HMGS* was found in above-ground tissues, including the stems, leaves, and flowers but lowest in the roots. Similarly, the constitutive high-level expression of *GbHMGS1* in ginkgo was found in the leaves and fruits because the aboveground organs function in terpenoid production and protection from solar radiation. On the contrary, the basic-level expression of *GbHMGS1* was found in the roots, which might be because the underground organs function in terpenoid production to prevent microbial infection or nutritional stress. One unexpected observation was that *GbHMGS1* had the lowest expression in female flowers. Alex et al. [46] studied the developmental expression pattern of *HMGS* and found that *HMGS* expression is the highest at early stages in the flower, seed and the seedling of *B. juncea*. Multiple homologous *HMGS* genes may participate in terpenoid biosynthesis in different tissues or during different developmental stages in one type of tissue. For instance, *HMGS1* and *HMGS2* from *H. brasiliensis* function as critical genes in the synthetic pathway of latex but are expressed in tissue-specific manner [38]. Our data revealed that *GbHMGS1* was a tissue-specifically expressed gene, suggesting that *GbHMGS1* may also participate in other unknown functions.

### 3.3. Transcript Level of GbHMGS1 and TTL Content Changes under Different Elicitor Treatments in Ginkgo Seedlings

Plant *HMGS* genes participate in the resistance of plants to adverse environmental stresses, and many plants exhibit a level of tolerance when exposed to extreme temperature for a period of time. A low temperature could up-regulates many genes, including *GbPAL* [47], *GbANS* [48], and G*bFLS* [49] that are involved in the flavonoid biosynthesis in *G. biloba*. However, few studies have investigated the relationship between cold stress and terpenoid biosynthesis. In the present study, *GbHMGS1* was markedly up-regulated by cold treatment. The up-regulated expression pattern of *GbHMGS1* by cold treatment confirmed its role in the response to low temperature stress.

Plants encounter a variety of abiotic stresses from the environment in their life. Among these stresses, UV-B stress is a critical environmental factor that threatens plant development and growth because the levels of UV-B on the surface of the earth increase continuously due to the decreased ozone layer. Plants have developed various metabolites, including UV-absorbing phenolic compounds and terpenoids, to protect against the deleterious effects of UV-B stress. Ultraviolet irradiation has been found to induce the expression of genes (e.g., *CAD* and *CHI)* [50,51]. However, few information concerning HMGS enzymes under UV stress is available. In the present study, UV-B treatment induced a steady up-regulation of *GbHMGS1* transcription during 48 h treatment, except for a decrease at 24 h in *G. biloba*. Therefore, delving into the regulation mechanism of *GbHMGS1* gene products may provide new insights into the processes regulated by UV-B.

MJ and SA are important phytohormones and signal molecules that regulate the response of plants against various stresses, including ozone exposure, UV radiation, and herbivore and pathogen attacks [52]. MJ and SA are elicitors that trigger the pathway of secondary metabolism in plants [53,54]. SA is also a key signal molecule in the systemic acquired resistance (SAR) of plants [55]. The exogenous application of SA has been conducted to enhance the biosynthesis of GA, GB, and bilobalide in *G. biloba* cell cultures [56]. Meanwhile, a set of MEP pathway genes, including *GbIDS2* [57], *GbDXS* [23] and *GbCMK2* [8], that are involved in ginkgolide biosynthesis in *G. biloba* were induced, they were all positively responsive to SA treatment. Furthermore, SA could up-regulate *HMGS* expression in *G. lucidum* [20], *S. miltiorrhiza* [31], *C. acuminata* [32], *T. wilfordii* [33], and *C. nobile* [34]. Similarly, the expression of the putative *GbHMGS1* was also strongly induced by SA in *G. biloba* (Figure 7E). Thus, *GbHMGS1* may be a potentially active member in SAR and participate in the synthesis of terpenoids, especially sesquiterpene for defending against local pathogen attack. Moreover, the SA-induced increase in TTL contents in the present study might be attributed to an integrated effect of a cluster of genes related to TTLs biosynthesis.

A number of reports have been published on the relationship between MJ and terpene metabolism [53,58]. Exogenously applied MJ can induce secondary defense metabolites along with the corresponding synthases in a wide range of plant species. For instance, MJ treatment enhanced the production of the mono- and diterpenoids and their respective terpene synthase activities in *Picea abies* [59,60]. In *S. miltiorrhiza*, MJ treatment enhanced the production of diterpenoid tanshinone and its terpene synthase activities [54,61]. In the present study, the accumulation of TTLs and the *GbHMGS1* transcription were up-regulated by MJ (Figure 7E,F). Nevertheless, MJ is not a specific inducer of the *GbHMGS1* gene because it also induces other genes. In addition, a previous study has shown that ginsenoside accumulation is far more raised by combining the elicitation of Eth and MJ than by single MJ elicitation, without causing any apparent growth inhibition [62]. This finding provides a new insight into the high accumulation of TTLs by applying diverse synergistic elicitors at the optimal concentration ratio.

Ethylene is a powerful natural regulating substance in plant metabolism and regulates many processes, including seed germination, root growth, fruit ripening, and many kinds of stress phenomena, during plant growth and development. Ethylene also modulates the expression of terpenoid biosynthesis-related genes, including *GbLPS*, *GbMVD*, *GbHMGR*, *GbDXS*, and *GbGGPPS* genes in *G. biloba* [63], by acting and interacting with other recognized plant hormones in trace amounts. The present study is the first to report that Eth increased TTL contents and up-regulated *GbHMGS1* expression in *G. biloba* (Figure 7E,F). Addicott et al. [64] found that temperature influences the tissue response to ethylene, and applying Eth at low temperatures significantly lowers the regulatory effect on the tissue. Additionally, Bae et al. [62] found that Eth at 50 μM enhanced both root growth and ginsenoside accumulation in ginseng adventitious root cultures. Therefore, we speculate that the transcript level of *GbHMGS1* may be improved by applying Eth at a proper high temperature, or the selection of the appropriate Eth concentration may further increase the TTLs content.

Considering the upregulated expression pattern of *GbHMGS1* under induction of all the tested elicitors (Cold, UV-B, MJ, SA, and Eth), we suggest that *GbHMGS1* is an elicitor-responsive gene, which participate in the stress resistance of ginkgo plant to adverse stresses and the signaling pathway involved in the regulation of exogenous hormones. However, the induction effects of various elicitors were diverse, which might be associated with many factors, such as the species of the elicitor, treatment concentration and time, the growth state of tissues, and different action mechanisms.

Accumulation of TTLs was enhanced by abiotic stresses and hormone treatments, may be associated with the upregulated expression of the genes in the MEP pathway, in light of the fact that the MVA pathway minimally contributes to TTL biosynthesis. For instance, Ethylene modulates the expression of terpenoid biosynthesis-related genes, including *GbDXS* [23], and the transcripts of *GbCMK2* [8], *GbDXS2* [23], and *GbIDS2* [59] are also positively responsive to SA and MeJA treatments. Therefore, further work will be carried out to clarify the relationship between the TTLs biosynthesis and all gene expression involved in the MEP pathway in *G. biloba*.

## 4. Materials and Methods

### 4.1. Plant Materials and Multiple Stress Treatments

The plant materials harvested from 27 year-old grafts of *G. biloba* cultivar “Jiafoshou” grown in the Botanical Garden of Yangtze University (Jingzhou, China) were used for gene cloning and tissue expression from roots, stems, leaves, young fruits, male flowers, and female flowers. All materials were frozen immediately in liquid nitrogen and stored at −80 °C prior to total RNA extraction.

One year-old seedlings of *G. biloba* cultivar “Jiafoshou” grown in a greenhouse were used as exogenous stress test materials. The growth conditions were set as follows: temperature, 23 ± 0.5 °C; humidity, 60–70%; light, 100 μmol·m^−2^·s^−1^; lighting time, 12 h day/12 h night; and watering with 100 mL of liquid fertilizer (containing 0.36 g N, 0.12 g P, and 0.14 g K) weekly. Ginkgo seedlings at 4–5 leaf stages were subjected to treatments with cold, UV-B, MJ, Eth, and SA, and three biological replicates were conducted for each sampling time point under various stress conditions. For UV-B treatment, the seedlings were exposed to UV-B (1500 μJ/m^2^) irradiation in a closed chamber, whereas the control was placed in a dark-closed chamber, leaves of both the treated and control seedlings were sampled at 0, 8, 16, 24 and 48 h. For cold stress, the seedlings were placed in a 4 °C illuminating incubator (400 μmol^−2^·s^−1^) without changing the other growth conditions. The control was treated at 25 °C light growth chamber. For exogenous hormone treatments, 100 μM MJ, 10 mM Eth, and 10 mM SA containing 0.01% (*v*/*v*) Tween 20 were sprayed onto the leaves of ginkgo seedlings while all other aspects of the growth conditions were similar to those of the controls, which are the plants treated with 0.01% (*v*/*v*) Tween 20 as control. Leaves from the treated and control plants were collected on the 0, 2nd, 4th, 6th and 8th days after the cold and exogenous hormone treatments. All leaves of test ginkgo seedlings were frozen immediately in liquid nitrogen after sampling, followed by storage at −80 °C for the analysis of the gene transcription level and TTL contents.

### 4.2. Cloning of the Full-length cDNA of GbHMGS1

Total RNA was extracted from the frozen leaves of *G. biloba* using the CTAB method described by Cai et al. [65]. The quality and concentration of the RNA were all determined by agarose gel electrophoresis and spectrophotometer analysis. The extracted RNA was reverse transcribed into 5′-RACE-Ready cDNA and 3′-RACE-Ready cDNA using PowerScript^TM^ Reverse Transcriptase (Clontech, Palo Alto, CA, USA) according to the Kit User Manual. A pair of degenerate primers HMGS-FP1 and HMGS-RP2 was designed based on the conserved amino acid and nucleotide sequences of the plant *HMGS* genes to obtain the internal conserved fragment by using the one-step RT-PCR kit (Dalian TaKaRa, Dalian, China) under the following conditions: 94 °C for 3 min; 32 cycles of amplification at 94 °C for 30 s, 54 °C for 30 s, and 72 °C for 2 min; and extended at 72 °C for 10 min. The amplified PCR products were purified using AxyPrep^TM^ DNA Gel Extraction Ki, ligated into the pMD18-T vector (Takara, Dalian, China), and introduced into *Escherichia coli* TOP10. Positive clones were selected and sent to Shanghai Sangon Biotechnology Company (Shanghai, China) for sequencing. Subsequent BLAST results confirmed that the amplified product was a partial fragment of the *HMGS* gene. Based on the sequence of the cloned *HMGS* fragment, the specific primer pairs (HMGS-5GSP1, HMGS-3GSP1) and the nested primer pairs (HMGS-5GSP2, HMGS-3GSP2) were designed to amplify the 5′ and 3′ ends of the cDNA of the *GbHMGS1* gene using the SMART^TM^ RACE cDNA Amplification Kit (Clontech, Palo Alto, CA, USA). 5′-RACE-PCR and 3′-RACE-PCR were performed according to the manufacturer's instructions. The PCR products were purified and cloned into the pMD18-T vector for sequencing. After comparing and aligning the sequences of the 5′ RACE and 3′ RACE, a pair of primers HMGSES1 and HMGSET2 was designed based on the stitching sequence of 5′ RACE, 3′ RACE, and the internal region products for amplifying the full-length cDNA sequence of *GbHMGS1* using 3′-Ready cDNA as the template. PCR was performed under the following conditions: 94 °C for 3 min, followed by 32 cycles of amplification (94 °C for 30 s, 56 °C for 30 s, and 72 °C for 2 min), and an extension for 10 min at 72 °C. After sequencing, the full-length cDNA of *GbHMGS1* was analyzed for molecular characterization. The sequences of all primers in the present study were shown in in Table S1 of the Supplementary Material.

### 4.3. Bioinformatics and Molecular Evolution Analysis

*GbHMGS1* gene sequences were translated into amino acid sequences using DNAMAN software. The open-reading frame (ORF) of the *GbHMGS1* gene was predicted using Vector NTI 11.5. Protein homology searches were performed using the NCBI BLAST server (http://www.ncbi.nlm.nih.gov/BLAST/). Multiple sequence alignment was performed with the software Vector NTI11.5 program. Compute pI/Mw tool (http://web.expasy.org/protparam/) was used to predict the molecular weight and isoelectric point (pI). The comparative modeling of the 3D structure of *GbHMGS1* was generated with SWISS-MODEL. The 3D structural analyses were performed with Weblab Viewerlite. A phylogenetic tree was constructed using HMGS protein sequences with neighbor-joining method using Clustal X2.0 and MEGA5.0 with 1000 bootstrap replicates.

### 4.4. Prokaryotic Expression and In Vitro Enzyme Activity Analysis of GbHMGS1

A pair of primers, HMGSYS2 and HMGSYT3, incorporated with restriction enzyme sites (*Eco* RI and *Hind* III, respectively) and protective bases were designed and synthesized to amplify the coding region of *GbHMGS1* by RT-PCR and clone the coding region into an expression vector pET-32a(+), yielding His-tagged *GbHMGS1* (pET32a-GbHMGS1). After confirmation by sequencing, the resulting recombinant plasmid was introduced into the *E. coli* strain BL21 (DE3) using heat shock method. A single colony of *E. coli* BL21 (DE3) cells harboring the expression plasmid pET32a-GbHMGS1 was inoculated in Luria–Bertani (LB) medium at 37 °C containing kanamycin (50 mg/L) and grown with 150 rpm shaking at 37 °C until the OD600 reached 0.6. For induction, the cells were induced by isopropyl-β-d-1-thiogalactopyranoside at a final concentration of 0.5 mM and further cultured at 30 °C for 3 h. The cells were lysed by sonication for 10 s at 4 °C and centrifuged at 25,000× *g* for 15 min. The supernates (soluble fraction) and pellets were examined by SDS-PAGE analysis, followed by Coomassie Brilliant Blue G250 staining. The recombinant GbHMGS1 protein from the induced cells was purified using Nickel-CL agarose affinity chromatography (Bangalore Genei, Bangalore, India) and used for in vitro enzyme activity determination.

The protein content was estimated by the Lowry assay [66] using bovine serum albumin as the standard. HMGS activity was determined by a modified method as described by Suvachittanont and Wititsuwannakul [18]. For the kinetics of acetoacetyl-CoA condensation by GbHMGS1, various concentrations of the substrate (20, 40, 60, 80 and 100 μM acetoacetyl-CoA) were added to 500 μL of the reaction buffer. A Michaelis–Menten (*V*_0_ = *V*_max_[S]/(*K*_m_ + [S], *R*^2^ = 0.99) curve was drawn based on the data. The specific activity, *K*_m_, and *V*_max_ of the recombinant GbHMGS1 protein were calculated using DPS ver. 9.5. (DPS Software Inc., Hangzhou, China).

### 4.5. Functional Complementation of GbHMGS1 in Yeast

The coding regions of the GbHMGS1 were amplified via PCR with the following two pairs of primers: HMGS-YS2 and HMGS-YT3. The forward primers contained the Bam HI restriction site, and the reverse primers contained the Xba I restriction site. The amplified products and the pYES2 vector (Invitrogen, Carlsbad, CA, USA) were digested with Bam HI and Xba I, and then ligated and transformed into *E. coli* DH5α. The constructed pYES2-GbHMGS1 plasmid was extracted from positive clones and subsequently transformed into YSC6274 with the Frozen-EZ Yeast Transformation II Kit (Zymo Research, Orange, CA, USA). The transformants were spotted on SC (-Ura) medium (6.7% yeast nitrogen base without amino acid; 2% galactose). Positive clones were further confirmed through PCR. The transformed diploid cells were induced to sporulate and form haploid cells containing pYES2-GbHMGS1. The diploid *S. cerevisiae* strain YSC1021 and the haploid *S. cerevisiae* strain YSC6274 lacking the HMGS allele were purchased from the Open Biosystems Yeast Knock-out Strain Collection (Open Biosystems, Huntsville, AL, USA). The diploid *S. cerevisiae* strain YSC1021 and haploid strain YSC6274 cells were separately grown on YPD (1% yeast extract, 2% bacto-peptone, and 2% glucose) + G418 (Geneticin, Thermo Fisher Scientific Inc., Waltham, MA, USA) and YPG (1% yeast extract, 2% bacto-peptone, and 2% galactose) + G418 medium to observe their growth status.

### 4.6. Quantitative RT-PCR Analysis of GbHMGS1 Expression Levels

The transcription levels of *GbHMGS1* in different ginkgo tissues and in the treated leaf samples from the ginkgo seedlings collected at different time points after stress treatments were detected by qRT-PCR. Total RNA from each sample was extracted using the CTAB method. The PrimeScript^TM^ RT reagent Kit with gDNA Eraser was used with 500 ng of each total RNA to synthesize single-strand cDNA (Dalian TaKaRa). qRT-PCR was conducted using a Bio-Rad Mini Opticon^TM^ Real-time PCR Mini Cycler (BioRad, Hercules, CA, USA) with SYBR^®^ Premix Ex Taq^TM^ II Kit (Dalian TaKaRa) according to the method of Xu et al. [67]. The PCR program was performed as follows: 95 °C for 5 min, followed by 40 cycles of 95 °C for 10 s and 60 °C for 30 s, with the melting curves stage (95 °C, 15 s; 60 °C, 1 min; and 95 °C, 15 s). *G. biloba 18S* gene (GenBank accession No. D16448) was used as the internal control for the normalization of all reactions. The primers for *GbHMGS1* (GbHMGSRTS and GbHMGSRTA) and *Gb18S* (Gb18SF and Gb18SR) for qRT-PCR were designed using Primer Premier 5.0 (Premier Biosoft International, Palo Alto, CA, USA). The efficiency of these primers was investigated by applying primer melting curve analysis and gel electrophoresis. The two genes (*GbHMGS1* and *18S*) were analyzed simultaneously for each plant sample. *GbHMGS1* expression was relatively quantified by calibrating its transcription level against *18S*. For the post-abiotic treatment analysis of *GbHMGS1* expression at different time points, the transcription levels of *GbHMGS1* in the treated ginkgo seedlings were compared with those of the controls to obtain the induction level of *GbHMGS1*. Each sample was run in triplicate and repeated thrice. 2^−ΔΔ*C*t^ was calculated to analyze the relative expression level of genes [68]. qRT-PCR data were technically replicated with error bars, representing mean ± SE (*n* = 3).

### 4.7. Determination of TTL Content in Ginkgo under Induction of Abiotic Stresses

Ginkgo seedlings were collected and subjected to freeze-drying. Ginkgolide A (GA), ginkgolide B (GB), ginkgolide C (GC), and bilobalide (BB) were extracted and quantified using gas chromatography (GC-14C, Shimadzu, Kyoto, Japan) with a wide bore capillary column [69]. The content of TTLs was calculated as the sum of the contents of GA, GB, GC, and BB and was expressed as dry weight (DW) percentages. All tests were conducted in triplicate, and data were presented as mean ± SD (*n* = 3).

### 4.8. Statistical Analysis

Data were analyzed with one-way ANOVA using SPSS 11.0 for Windows (SPSS Inc., Chicago, IL, USA). Comparisons between multiple treatment groups were performed with Tukey’s honestly significant difference test. *p* < 0.05 was considered to be statistically significant.

## 5. Conclusions

This study first isolated and characterized *GbHMGS1*-encoding HMGS enzyme in the MVA pathway from *G. biloba*. Multiple alignments showed that the deduced *GbHMGS1* was highly identical to other HMGSs and contained all the conserved motifs and conserved sites of HMGSs. Prokaryotic expression and functional complementation assays revealed that *GbHMGS1* encoded a functional HMGS and could catalyze the conversion of acetoacetyl-CoA to HMG-CoA involved in MVA pathway. The transcript level of *GbHMGS1* preferentially expressed in leaves of *G. biloba*. The *GbHMGS1* expression under different elicitor treatments (cold, UV-B, MJ, Eth, and SA) in ginkgo seedlings further verified the crucial roles of the *GbHMGS1* in stress response and sesquiterpenoid biosynthesis. Therefore, the present work on *GbHMGS1* is a useful supplement to elucidate the overall molecular regulatory mechanism underlying terpenoid especially sesquiterpene biosynthesis in *G. biloba*.

## Figures and Tables

**Table 1:** The enzyme activity determination of recombinant GbHMGS1.

Enzyme	*k*_cat_ (min^−1^)	*K*_m_ (μM)	*k*_cat_/*K*_m_ (μM^−1^·min^−1^)	Specific Activity (μmol·min^−1^·mg^−1^)	*V*_max_ (nM·min^−1^)
No HMGS in pET32	ND	ND	ND	3.4 ± 0.5	ND
GbHMGS1	195.4 ± 13.1	689 ± 32.6	0.28 ± 0.01	15.5 ± 4.8	37.1 ± 5.4
HvHMGS	ND	530 ± 50	ND	41.3 ± 2.9	130 ± 30

ND, not determined.

## Supplementary Materials

Supplementary data associated with this article can be found in the online version.
