# Supplementary material for: Characterization, Function, and Transcriptional Profiling Analysis of 3-Hydroxy-3-methylglutaryl-CoA Synthase Gene (GbHMGS1) towards Stresses and Exogenous Hormone Treatments in Ginkgo biloba
Source: Molecules. 2017 Oct 12;22(10):1706. doi: 10.3390/molecules22101706 (PMC6151752; doi:10.3390/molecules22101706)

Table S1. Primers used in this study.

Name	Sequence (5'-3')	Description
HMGS-FP1	GARGTSGGNACNGARAC	Degenerate primer, forward
HMGS-RP2	TCNGGYTTRTARAARTCRTA	Degenerate primer, reverse
HMGS-3GSP1	GGATGCCCTGGAAACCTTTGATGGAGTA	Forward primer for 3' RACE, outer
HMGS-3GSP2	TTGGGATGGGCGATATGGTCTCGTTGT	Forward primer for 3' RACE, nested
HMGS-5GSP1	GGGTACTCGCTGGCTAAATTGGGCTTAT	Reverse primer for 5' RACE, outer
HMGS-5GSP2	GAAACAACGAGACCATATCGCCCATCCC	Reverse primer for 5' RACE, nested
HMGSES1	ATGGCGTCTCATCCAGAAAAT	Forward primer for OPF-PCR
HMGSET2	TTACAATTGATCATGGATGATACC	Reverse primer for OPF-PCR
HMGSYS2	GGAATTCATGGCGTCTCATCCAGAAAAT	Primer for *E. coli*-PCR, forward
HMGSYT3	CAAGCTTTTACAATTGATCATGGATGATACC	Primer for *E. coli*-PCR, reverse
HMGS-YS2	CGGGATCCATGGCGTCTCATCCAGAAAAT	Primer for Yeast-PCR, forward
HMGS-YT3	CTCTAGATTACAATTGATCATGGATGATACC	Primer for Yeast-PCR, reverse
Gb18SF	ATAACAATACTGGGCTCATCG	Primer for qRT-PCR, forward
Gb18SR	TTCGCAGTGGTTCGTCTTTC	Primer for qRT-PCR, reverse
GbHMGSRTS	GCCAAAGCAAGTAGGCAACA	Primer for qRT-PCR, forward
GbHMGSRTA	ATGCTGACCCTCCCGAATT	Primer for qRT-PCR, reverse

Table S2. Protein sequence of GbHMGS1 similarity to the HMGS proteins from other plant species.

**Species**	**Accession No.**	**Identity (%)**
Taxus x media	AAT73206.1	85%
Pinus sylvestris	CAA65250.1	83%
Narcissus tazetta	AHF81872.1	79%
Sorghum bicolor	KXG40334.1	78%
Theobroma cacao	XP_007040101.2	77%
Hevea brasiliensis	AAK73854.1	75%
Camptotheca acuminata	ACD87446.1	74%

Figure S1. The nucleotide acid sequence and deduced amino acid sequence of *GbHMGS1*. The initial codon and the stop codon are highlighted in red square box.

1 GGGACAGACAAGTTTGTTCTCCATATTCGAGGGTTTGCTATAAGCTGCCAGCCCCTGTGCTTCTCCTAAAGAACAGATTTGGCTGGTTTG

91 TGCAGAAAATGGCGTCTCATCCAGAAAATGTTGGTATTTTGGCTATGGATATCTACTTTCCGAGCACATGTGTTCAGCAGGATGCCCTGG

M A S H P E N V G I L A M D I Y F P S T C V Q Q D A L

181 AAACCTTTGATGGAGTAAGTAAAGGGAAATACACAATTGGCCTTGGACAAGACTGCATGGCATTTTGCATGGACTTGGAAGATGTGATTT

E T F D G V S K G K Y T I G L G Q D C M A F C M D L E D V I

271 CCATGAGCTTGACTGTAGTATCCTCACTCTTGGACAAGTATGGAATCGATCCAAAAAGAATTGGTCGCTTAGAAGTTGGCAGTGAAACTG

S M S L T V V S S L L D K Y G I D P K R I G R L E V G S E T

361 TTATTGACAAGAGCAAATCCATAAAGACCTGGTTGATGCAGATTTTTGAGGAATGTGGCAATAGTGAGATTGAAGGTGTGGACTCAACAA

V I D K S K S I K T W L M Q I F E E C G N S E I E G V D S T

451 ATGCATGCTATGGAGGAACTGCAGCTCTGCTTAACTGTGTTAACTGGGTTGAAAGCAGGTCTTGGGATGGGCGATATGGTCTCGTTGTTT

N A C Y G G T A A L L N C V N W V E S R S W D G R Y G L V V

541 CTACAGACAGTGCAGTCTATGCTGAAGGTCCAGCCCGACCTACAGGGGGAGCAGCTGCTGTTGCTATGCTGATAGGGCCCAATGCACCTA

S T D S A V Y A E G P A R P T G G A A A V A M L I G P N A P

631 TAGTGTTTGAAAGCAAATACAGGGGAACACACATGTCTCATGCGTATGACTTTTATAAGCCCAATTTAGCCAGCGAGTACCCAGTTGTGG

I V F E S K Y R G T H M S H A Y D F Y K P N L A S E Y P V V

721 ATGGGAAGCTTTCACAAACTTGCTATCTCATGGGACTGGACTCATGCTACAAACGGTTTTGTAATCGGTTTGAGGAGGGAGAAGGAAGAC

D G K L S Q T C Y L M G L D S C Y K R F C N R F E E G E G R

811 AATTTTCTCTTTCAGATGCAGATTATGTAGCATTTCACTCTCCATACAATAAGCTTGTGCAAAAGAGCTTTGCTCGACTGTTGTTCAATG

Q F S L S D A D Y V A F H S P Y N K L V Q K S F A R L L F N

901 ATTTCTCAAGACATGCCAGTTCTGTTGGGAAGGATGCGCAAGAAAAGCTAGAGCCGTATGCAGGCTTGTCTGATGAAGATAGCTACAGTA

D F S R H A S S V G K D A Q E K L E P Y A G L S D E D S Y S

991 GCCGTGAACTAGAAAAGGTTTCTCAGCAGGTTGCTAGGCCATTGTATGATGTAAAAGTTCAACCATCAACCTTATTGCCAAAGCAAGTAG

S R E L E K V S Q Q V A R P L Y D V K V Q P S T L L P K Q V

1081 GCAACATGTACACAGCATCTCTTTATGCAGCGTTTGCCTCTATTATACATAACGAGCATGATACCCTGGAGGGGCAGAGGGTGTTGATGT

G N M Y T A S L Y A A F A S I I H N E H D T L E G Q R V L M

1171 TTTCTTATGGGAGTGGTTTGGCTTCCACAATGTTTTCTTTGAAAATTCGGGAGGGTCAGCATCCTTTCACCTTAACAAATATTGCAGATG

F S Y G S G L A S T M F S L K I R E G Q H P F T L T N I A D

1261 TTATGGATGTCTCGAGCAAGCTTGATTCTCGACATGTGTTGTCCCCTGAAGATTTTATGGTCAACATGAAGCTGATGGAGACTCTATATG

V M D V S S K L D S R H V L S P E D F M V N M K L M E T L Y

1351 GAGCAAAAGATTTCATTTCATGCTCTGATCACAACTTGCTGCGATCTGGAACATTTTATTTGACTCAAGTAGATTCAATGTACCGACGTT

G A K D F I S C S D H N L L R S G T F Y L T Q V D S M Y R R

1441 TCTATGCCCAGAAGGTTGCAAGTCCAGATGACACTCATGAGAAGGTCAAACTTGCAAATGGTATCATCCATGATCAATTGTAACACATGT

F Y A Q K V A S P D D T H E K V K L A N G I I H D Q L *

1531 AGATTGAGAGATTGAGTAGTATCACATCTACATATCATGTGCAGGAGAGGTTGAATGTAAGTTCTGCATTGTAACATAGCATTCTTGAGC

1621 CTCCCCCTTCATCTTTTTGGTAGCTCTTTTCCTTCATTTTAAGTGGCATGATGGCATTGAGAGAATAAATTGGGAGAATGCTATTCAGGT

1711 AATTATTGCTCGAGCATGCATTGGCCTCCTTGTTCTTTGTCAATTGTGGAATAAACATTGGCAAAGGAACTCTTGCCTTTTTTACTGCAT

1801 TCCGAAATCTATCCATCTGCTGATGTTTGGAGAGGATTTAAGGAAGCATTTCTTCAGAAATGAACTAGTATTTATGGGTCTTTGTATCTA

1891 AATTAAATCCTTTGGTTTCTGTTTGCCAAAAAAAAAAAAAAAAAAAAAAAAAAAA

Figure S2. SDS-PAGE analysis of recombinant GbHMGS1 protein in *E. coli* BL21 (DE3). After IPTG induction, *E. coli* BL21 cells containing pET32a-GbHMGS1 vector were grown at 30 ℃ for 4 h. M, molecular marker; lane 1, total cellular protein with 1mM IPTG induction for 4h; lane 2, insoluble protein with 1mM IPTG induction for 4h; lane 3, soluble protein with 1mM IPTG induction for 4h; P, purified recombinant GbHMGS1 protein with IPTG induction for 4 h. Purification of the recombinant GbHMGS1 protein was achieved by Nickel-CL agarose affinity chromatography and used for enzyme activity assay.

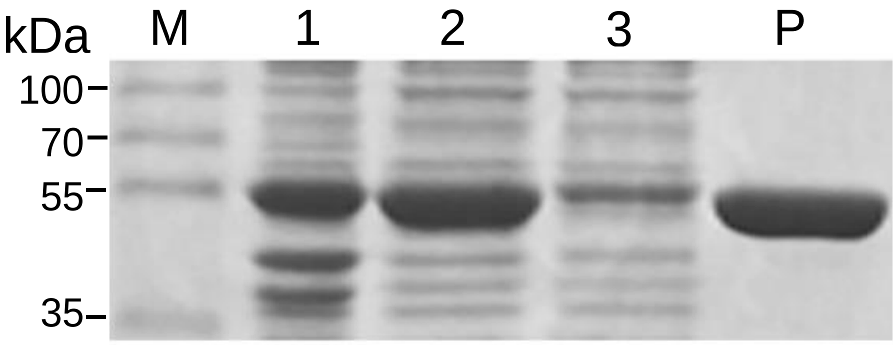

Supplement: Supplementary file 1 [file molecules-22-01706-s001.pdf]
